# Atrial Fibrillation in Elite Athletes: A Comprehensive Review of the Literature

**DOI:** 10.3390/jcdd11100315

**Published:** 2024-10-09

**Authors:** Christos Kourek, Alexandros Briasoulis, Elias Tsougos, Ioannis Paraskevaidis

**Affiliations:** 1 Department of Cardiology, 417 Army Share Fund Hospital of Athens (NIMTS), 11521 Athens, Greece; chris.kourek.92@gmail.com; 2 Medical School of Athens, National and Kapodistrian University of Athens, 15772 Athens, Greece; alexbriasoulis@gmail.com; 3 Department of Cardiology, Hygeia Hospital, 15123 Athens, Greece; cardio@tsougos.gr

**Keywords:** atrial fibrillation, elite athletes, pathophysiology, management

## Abstract

Although the benefits of exercise training have been shown repeatedly in many studies, its relationship with the occurrence of atrial fibrillation (AF) in competitive athletes still remains controversial. In the present review, we sought to demonstrate a comprehensive report of the incidence, pathophysiology, and therapeutic approaches to AF in elite athletes. A 2 to 10 times higher frequency of AF has been shown in many studies in high-intensity endurance athletes compared to individuals who do not exercise. Moreover, a U-shaped relationship between male elite athletes and AF is demonstrated through this finding, while the type and the years of physical activity seem to relate to AF development. A strong correlation seems to exist among the type of exercise (endurance sports), age (>55 years), gender (males), and the time of exercise training, all contributing to an increased risk of AF. The pathophysiology of AF still remains unclear; however, several theories suggest that complex mechanisms are involved, such as bi-atrial dilatation, pulmonary vein stretching, cardiac inflammation, fibrosis, and increased vagal tone. Elite athletes with AF require a comprehensive clinical evaluation and risk factor optimization, similar to the approach taken for nonathletes. Although anticoagulation and rate or rhythm control are cornerstones of AF management, there are still no specific guidelines for elite athletes.

## 1. Introduction

Regular exercise training has shown excellent results in the battle against traditional cardiovascular risk factors, and has demonstrated an improvement in insulin sensitivity, lipidemic profile, and all-cause mortality, with advanced levels of endurance training further improving these outcomes [1,2,3]. Although the benefits of exercise training have been repeatedly shown in many studies, its relationship with the occurrence of atrial fibrillation (AF) in competitive athletes still remains controversial.

AF is one of the most common cardiac arrhythmias observed in middle-aged athletes [4]. Recent studies have presented a significant association between regular endurance exercise and an increased risk of AF, not only in elite athletes, but also in non-elite athletes who participate in vigorous exercise [5,6,7,8]. Despite exercise’s favorable effect on various traditional risk factors of arrhythmogenesis, an increase in physical activity only modestly reduces incident atrial fibrillation. On the other hand, approaching the extreme limit of exercise activities, endurance athletes, who participate in intense exercise training programs, demonstrate higher AF risk [9].

“Athlete” is a term that derives from the Greek word “athlos” which means achievement. Athletes are “individuals of young and adult age, either amateur or professional, who are engaged in exercise training on a regular basis and participate in official sports competition”, according to the European Society of Cardiology (ESC) [10,11], while elite athletes are “athletes who participate at the highest level of national or international competitions such as the Olympic Games (>10 h/wk)” [10,11].

In the present review, we sought to demonstrate a comprehensive report of incidence, pathophysiology and therapy management of AF in elite athletes.

## 2. Epidemiology and Predisposal Factors

**Figure 1:**
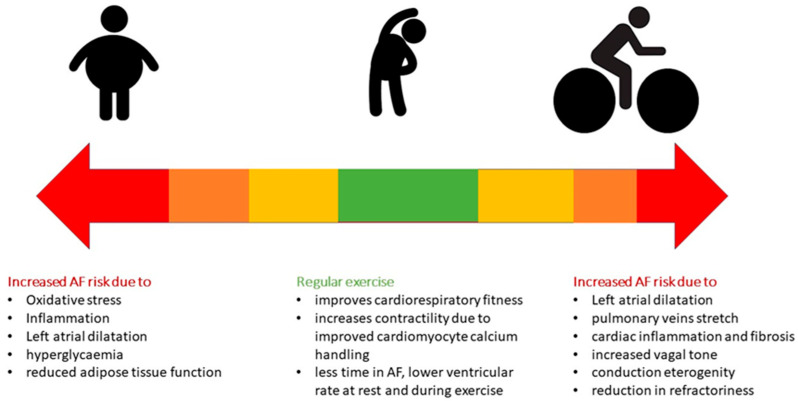
Atrial fibrillation (AF) risk varies across the spectrum of exercise training. Approaching the extreme borders of physical activities (minimum physical activity at the one border and endurance sports at the other), there is an increase in AF incidence.

Many retrospective observational studies and meta-analyses have estimated a 2 to 10 times higher frequency of AF in high-intensity endurance athletes compared to individuals who do not exercise [6,7,8,12,13,14,15,16,17]. A U-shaped relationship between male elite athletes and AF is demonstrated through this finding, suggesting that both the type (endurance) and the dose (hours of training) of physical activity seem to relate to AF development [14,18,19,20,21]. This U-shaped relationship has not been confirmed in women [14]. A recent meta-analysis showed that physical activity has a dose-dependent J-shape effect on AF risk, with increased risk at very low and very high levels of physical activity [15]. This effect seems to be gender-specific and more pronounced in younger males. Regular exercise of either low or moderate intensity seems serve as a preventive strategy for cardiovascular disease and AF, while higher-intensity endurance exercise may lead to AF [6,7,8,12,13,14,15,16,17,18,19,20,21]. Moreover, although the prevalence of AF increases with age, the relative risk for athletes compared to nonathletes seems to be higher in younger athletes [22,23]. A retrospective observational cohort study among young elite Spanish athletes (N = 6813) with a mean age of 22 years assessed the incidence of AF over a 20-year time period, and found it to be low (0.3%), even in endurance athletes, with only 21 out of 6813 athletes having AF (18 with paroxysmal, 1 with persistent, and 2 with long-standing persistent AF) during the 20-year study [24]. In the same study, there was an association between increased AF risk and years of exercise training, age, and left atrial (LA) anteroposterior diameter [24]. The prevalence of AF in competitive athletes ranges from 0.3% to approximately 13%, and differs according to the age and the modality of exercise training [13,25,26,27]. A large meta-analysis of 13 studies, including approximately 64,000 athletes, showed that athletes were 2.46 times more likely to have prevalent AF (95% CI 1.73 to 3.51). This odds ratio increased to 3.6 for athletes aged <55 years of age [25]. Athletes participating in mixed sports (such as football, basketball, and American football) have increased AF burden compared to those engaged in endurance sports (such as Nordic skiing, orienteering, and rowing; B = −0.5476, *p* = 0.0204) [25]. The risk of AF across the spectrum of exercise training is illustrated in Figure 1.

Data on differences in AF incidence between males and females are limited due to a lack of specific trials. Only retrospective studies with small sample sizes that have been conducted over the last 2 decades, which compared men and women, showed that AF incidence may be higher in female endurance athletes compared to males [28,29]. Specifically, Wilhelm et al. enrolled 61 female and 60 male athletes and found that, for a comparable amount of training and performance, male athletes showed more pronounced atrial remodeling, a concentric type of ventricular remodeling, and an altered diastolic function [30]. Another study by Sanchis et al. presented gender-related differences in the adaptation of atrial performance to training, with women exhibiting greater bi-atrial deformation and smaller right atrial size compared to men [31].

An association among the type of exercise (endurance sports), age (>55 years), gender (males), and the duration of exercise training with increased AF risk has been proven through studies [24]. Another significant clue regarding the AF spectrum is the strong association between cardiomyopathies—including dilated, hypertrophic, and arrhythmogenic cardiomyopathy—and AF in younger athletes (with the prevalence of AF ranging from 10% to 25% in each subtype), as well as the association between long QT syndrome (LQTS), Brugada syndrome, and AF [32]. Finally, studies indicate that the highest incidence of AF is observed in the Caucasian population compared to other racial groups [33,34].

AF may be the first manifestation of an inherited cardiac condition in young athletes. There is a strong association between AF and genes such as KCNA5, SCN5A and TTN, while genes like LMNA and KCNQ1 present a weaker association with AF [35,36,37,38].

Another predisposing factor for AF may be the consumption of sports supplements, which is common among individuals who exercise regularly, both at competitive and non-competitive levels. Non-approved anabolic steroids are being used at competitive levels in order to improve performance, and have been associated with an increased risk of AF in young athletes [39,40]. Other sports supplements that contain high concentrations of caffeine and stimulants, such as taurine and guarana, may cause cardiac arrhythmias after regular consumption, including AF, potentially by stimulating the autonomic system [41,42]. Moreover, high consumption of these supplements may lead to both an increase in blood pressure by 10 mmHg and an increase in heart rate by 5–7 beats per minute, even in healthy people [43].

## 3. Pathophysiology

The pathophysiology and underlying mechanisms of AF in elite athletes still remain unclear. Theories suggest that complex mechanisms may contribute to AF, including alterations in autonomic tone, systemic inflammation, electrical remodeling, and LA enlargement and fibrosis [12,44,45,46,47,48,49,50].

Cardiac remodeling is the first proposed mechanism of AF. Mechanisms, including LA structural changes such as dilatation and pulmonary vein stretching, cardiac inflammation, fibrosis, and increased vagal tone, that may cause conduction heterogeneity and a reduction in refractoriness are responsible for AF development in endurance athletes [51]. Athletes present increased arterial blood pressure during exercise and in combination with atrial wall stretching after long-term strenuous endurance training, which may lead to the development of arrhythmogenic areas due to microtrauma, inflammation, and fibrosis [13]. Furthermore, there is an association between endurance exercise and both bi-atrial and ventricular enlargement [52]. Specifically, LA enlargement, both in size and volume, which is associated with an increased risk of AF, is present in approximately 20% of young competitive athletes [13,50]. An observational study involving 492 marathon runners reported a correlation between hours of training and LA enlargement, indicating a higher risk of AF in athletes with more hours of exercise training (24% in <1500 h, 40% 1500–4500 h and 83% in >4500 h) [6].

Fibrosis and its potential role in exercise-induced AF is still under investigation. A study including 45 veteran elite athletes showed increased markers of myocardial fibrosis, such as carboxyterminal telopeptide of collagen type I (CITP 5.4 vs. 2.9 microg/L, *p* < 0.001), plasma carboxyterminal propeptide of collagen type I (PICP 259 vs. 166 microg/L, *p* < 0.001), and tissue inhibitor of matrix metalloproteinase type I (350 vs. 253 ng/mL, *p* = 0.01), compared to sedentary controls [53]. Similar findings of myocardial fibrosis were presented a few years later in another study involving 12 veteran male endurance athletes who were assessed by cardiac magnetic resonance imaging (MRI) [54]. Specifically, half of them (6/12 athletes) demonstrated late gadolinium enhancement (LGE) compared to sedentary controls. The number of years spent in training (*p* < 0.001) and the number of competitive marathons participated in (*p* < 0.001) predicted prevalence of LGE on cardiac MRI. The proposed theory is that regular exercise can induce chronic systemic inflammation, as shown by increased levels of CRP, and may lead to atrial electrical remodeling and, therefore, to the development of AF [55]. However, whether AF can be treated with anti-inflammatory drugs remains controversial, and more evidence is required. In a retrospective cohort study based on male long-distance cross-country ski race participants and men from the general population, the authors evaluated the risk of AF and atrial flutter (AFl) depending on years of exercise [8]. It was shown that years of regular endurance exercise were significantly associated with an increased risk for both arrhythmias, with a 1.16 (95% confidence interval 1.06 to 1.29) times higher risk for AF and a 1.42 (95% confidence interval 1.20 to 1.69) times higher risk for AFl per 10 years of endurance exercise [8].

AF in athletes is considered to be vagal-mediated [7]. AF is triggered through the macro-re-entry pathway by an increase in the dispersion of the atrial refractory period via the activation of the iKach channel [56]. Moreover, most athletes present a lower baseline resting heart rate, which is also a predictor of AF [49,57]. Exercise-related increases in sympathetic tone in endurance athletes could also be predisposing factors for AF [58,59]. In general, the combination of increased basal vagal activity and adrenergic activation may serve as a predisposing factor for AF [12].

There is still no clear pathophysiological mechanism regarding the differential response to AF between male and female elite athletes. The most dominant hypothesis suggests that specific criteria in females, including smaller atria, shorter P-wave duration, differences in autonomic tone, and lower LV mass and wall thickness, may be the keys to these differences [17].

Electrolyte abnormalities may also be a possible trigger of AF in athletes involved in vigorous exercise. Vigorous-intensity activities are defined as those with an oxygen consumption of more than 6 METs, while endurance sports require repeated isotonic contractions of large skeletal muscle groups. Such athletic activities include running (>5 mph), cycling, swimming, shoveling, soccer, and cross-country skiing or speed skating among winter sports. These athletes may present dynamic fluid alterations which can lead to dehydration, changes in pH, and depletion of electrolytes, including Na, K, and Mg [51]. These biochemical changes can lead to supraventricular arrhythmias such as AF.

Finally, a less likely theory is that of acid reflux disease. Specifically, physical activity could induce gastroesophageal reflux, and therefore lead to AF [60]. The direct correlation between exercise and a decrease in intra-esophageal pH, as well as acid reflux disease and an increased risk of AF, has been shown in many observational studies in elite athletes [61,62,63,64]. Among 163,627 patients, acid reflux disease was shown to significantly increase the risk of AF by 39% [64].

**Figure 2:**
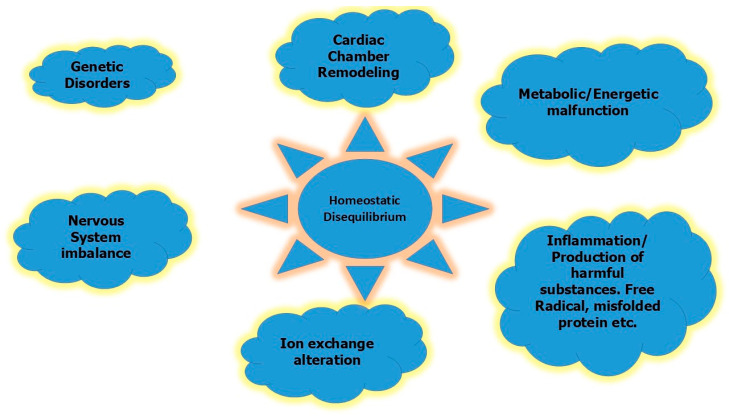
Presumed mechanisms of AF in elite athletes.

The pathophysiological mechanisms involved in AF in competitive athletes are demonstrated in detail in Figure 2.

## 4. Clinical Significance, Screening and Management

Physical activity seems to contribute to a decrease in all-cause cardiovascular mortality and ischemic stroke, as shown in the EORP-AF study [65]. This study included 2442 patients diagnosed with AF, separated into groups of mild-, moderate-, or high-intensity training, as well as controls without exercise. The intensity of physical activity was inversely correlated with the CHA2DS2-VASc score, indicating that a smaller percentage of patients in the high-intensity group required anticoagulation compared to those in the mild-intensity group, where the percentage of patients receiving anticoagulants was higher [65]. Moreover, the high-intensity exercise training group presented reduced mortality and progression rates to permanent AF compared to groups of mild intensity or no exercise [65].

Elite athletes may present higher levels of profibrotic markers which have been associated with incident or recurrent AF in the general population [66,67,68], including galectin-3 [69], the suppression of tumorigenicity 2 (ST2) [70], and microRNA-21 (miR-21) [71], as well as tissue inhibitors of metalloproteinase 1 (TIMP-1), C-terminal telopeptide of type I collagen (CITP), and procollagen type I carboxy-terminal propeptide (PICP) [53].

Clinical AF subtypes include AF secondary to underlying structural heart diseases (e.g., heart failure or valve disease), lone AF, and AF in athletes. Physical activity seems to limit the presence of AF symptoms and improve cardiac remodeling in people with cardiac structural disease. However, trials often fail to prove the positive effect of exercise training. In the HF-ACTION study, 193 individuals with AF and heart failure (HF) initially underwent 36 sessions of supervised exercise, and then continued a 2-year home-based rehabilitation program [72]. Investigators did not observe any difference in the number of hospitalizations, mortality, events related to AF, or other major outcomes between the training and the control group after a median follow-up of 2.6 years. In another equally significant study, the RACE 3 trial, 119 individuals with persistent AF and HF performed moderate intensity cardiac rehabilitation while 126 controls with the same background received the usual care. The trial showed that a sinus rhythm was present in 75% of patients who performed cardiac rehabilitation versus 63% in patients who received usual care after 1 year of follow-up [73]. The triggering of the sympathetic nervous system through acute exercise results in positive inotropic, chronotropic, and lusitropic effects on the myocardium. Regular training improves peak oxygen uptake, increases cardiac output, and therefore, improves functional capacity in all patients, regardless of whether they have an initial sinus rhythm or AF [74]. Another beneficial effect of exercise is improved cardiomyocyte calcium handling, which contributes to gradual hypertrophy and the increased contractility of the myocardium [75]. It is a general consensus that athletes and non-athletes who present AF should exercise on a regular basis after a comprehensive clinical assessment for underlying pathology and that guidelines should be more individualized.

In studies, such as the ARREST-AF cohort study or the ACTIVE-AF trial, regular exercise programs exhibited a reduction in AF-related symptoms, AF incidence, and AF recurrence rates [76,77]. In a meta-analysis, effects of different exercise modalities on quality of life in AF patients were evaluated [78]. In 12 studies, 670 participants underwent aerobic exercise, aerobic interval training (AIT), or other exercise modalities such as yoga, qigong, and cardiac rehabilitation protocols. All exercise modalities demonstrated statistically significant beneficial effects on general health and vitality, measured by the Short Form 36-item questionnaire. Specifically, cardiac rehabilitation protocols and AIT increased peak VO_2_, while AIT also significantly reduced AF burden. Moreover, qigong significantly improved the 6-min walk test in these individuals [78].

Preparticipation screening plays a crucial role in the early detection and management of AF in elite athletes who may be asymptomatic, thereby increasing the risk of stroke or other cardiovascular events. Early identification through screening can guide appropriate interventions, reducing the risk of serious complications. Athletes may benefit from more targeted, context-specific evaluations based on their activity levels. Although there is no established screening strategy, recommended tools for screening in athletes with palpitations include a 14-point medical history, a physical examination, and a 12-lead ECG [38]. A description of symptoms and their association with exercise, and any associated symptoms of lightheadedness, chest pain, or syncope, is important in order to diagnose a potential episode of AF. Moreover, a family history of cardiovascular disease increases the likelihood of underlying heart disease. Athletes aged 25 years or more may need further evaluation based on cardiovascular risk factors for atherosclerotic heart disease [79]. A physical examination revealing irregular heartbeats may raise suspicion of AF or premature beats. An ECG is essential for confirming the clinical suspicion of arrhythmias and can also aid in diagnosing other electrical or structural cardiac conditions [80]. As a result, it has become a routine part of sports medicine examinations. Distinguishing between benign ECG changes due to physiological adaptation and pathological changes is a critical skill for physicians involved in sports medicine. Regularly updated ECG guidelines help reduce false positives while maintaining good sensitivity and specificity [81]. However, the routine use of echocardiography is not widely adopted as a screening test, but it plays an important role in the evaluation of symptomatic athletes (i.e., palpitations, syncope, dyspnea) and athletes with known or suspected cardiac conditions [82].

Exercise stress tests may be useful screening methods for athletes with palpitations during exercise due to their ability to mimic the adrenergic activity that triggers the symptoms. In cases where this is not feasible, then a monitoring strategy that enables rhythm determination during the activity may be useful [38]. Smart devices which have the ability to record athletes’ rhythms, such as wearable smartwatches, other commercially available wearable devices, or handheld ECG recording devices, may be useful in recording the athlete’s heart rhythm during the time they are symptomatic [38]. Specifically, wearable technologies like smartwatches and fitness trackers (e.g., Fitbit, Apple Watch) offer continuous monitoring of heart rate and can help detect irregular rhythms, including AF. However, these devices have limitations, including accuracy issues and the necessity for the user to have the device always readily available [83,84]. Their accuracy can be affected by physical movement during activities, potentially leading to false positives or missed arrhythmias. As a result, while they are useful for initial screening, they should be followed up with medical-grade diagnostics for confirmation when abnormal readings occur. Finally, electrophysiology studies to exclude (concealed) accessory pathways and AVNRT, Ajmaline testing to rule out Brugada syndrome, potential genetic testing (e.g., SCN5A, LMNA), and cardiac magnetic resonance may be useful diagnostic techniques for excluding other arrhythmogenic conditions.

It is common policy that an athlete with a previous medical history of paroxysmal AF, but with a sinus rhythm in the ECG, can exercise without limitations. However, elite athletes in more competitive sports who present more than one episode of AF are advised to be more cautious as rate control cannot be assured during high-intensity training, even under medication [85]. In this case, catheter ablation for AF could be a promising solution for athletes who present AF symptoms. Younger and middle-aged athletes with paroxysmal AF are encouraged to reduce the intensity and the duration of their physical activity as an initial approach to limit exercise-induced AF [8,85]. However, due to the shortage of cumulating data, further studies with a longer follow-up of training effects are required for athletes and other clinical subgroups of AF patients in order to extract information and provide specific recommendations.

Asymptomatic athletes with AF and normal heart rates generally do not need rate control therapy [11]. However, for symptomatic athletes, the selection of rate control medications must be carefully tailored to the individual, considering their physical demands and the impact of different drugs on exercise performance. Endurance athletes with AF are shown to present more adverse symptoms than sedentary patients without exercise [65]. Athletes with symptoms including fatigue, dizziness and syncope—induced by rapid AV nodal conduction during physical activity—are recommended to terminate their physical activity and should be referred to physicians for better rate control [11]. Despite the negative impact of beta-blockers on athletes’ physical performance, they remain a common option among drug therapies [11]. Verapamil, a non-dihydropyridine calcium channel blocker, is a viable option and can sometimes be more effective than beta-blockers in controlling heart rate, especially in athletes who are sensitive to the fatigue or reduced exercise tolerance commonly associated with beta-blockers [86]. Verapamil slows conduction through the AV node without significantly impairing exercise capacity, making it a reasonable choice in selected patients. On the other hand, digoxin, which primarily affects resting heart rate by increasing vagal tone, is often less ideal for athletes because it has a minimal impact on controlling heart rate during exertion [11]. However, it may still have a role in certain cases, such as when an athlete has comorbid conditions (e.g., heart failure with reduced ejection fraction) or when resting rate control is particularly challenging [11]. The most potent solution in order to prevent athletes from developing sinus bradycardia at rest or chronotropic incompetence during training would be a combination of individually titrated negatively chronotropic agents [11].

Class I and class III antiarrhythmics are the main components in the armamentarium of the rhythm control in AF, but they have some limitations. On the one hand, class III antiarrhythmics, such as sotalol and amiodarone, may be insufficient for control or relatively contraindicated in young athletes, respectively [11]. On the other hand, the use of class I antiarrhythmics requires caution due to the potential risk of atrial flutter, but still can be a viable option in well-selected cases—especially in athletes with normal EF. The necessity of beta-blockers or cavo-tricuspid isthmus (CTI) ablation may not be mandatory in all cases [11]. Close monitoring and individualized treatment plans should be prioritized, balancing the risk of potential arrhythmias against the benefits of maintaining the sinus rhythm with antiarrhythmics. Prophylactic CTI ablation may not reflect the current standard of care unless atrial flutter is clearly documented. Class I antiarrhythmics could be used as a ‘pill-in-the-pocket’ approach in patients with sporadic AF [87].

Through clinical practice, rate control may often be ineffective in relieving symptoms for athletes given their higher baseline vagal tone and increased cardiovascular demands. Flecainide has its limitations, especially in highly active patients, and the need for symptom control often pushes clinicians toward considering ablation early in the treatment plan. Recent guidelines reflect this trend toward considering catheter ablation as a first-line option in symptomatic patients with AF, irrespective of athletic status, thus acknowledging the benefits of early ablation for symptomatic AF, particularly in patients who do not respond well to medical management or who prefer a non-pharmacological approach [38]. It could offer the potential for symptom relief and a return to peak athletic performance. Ablation techniques have advanced significantly, leading to higher success rates, especially when carefully selected based on a thorough evaluation of the athlete’s cardiovascular health. It is crucial to rule out underlying structural heart disease, cardiomyopathies, genetic predispositions, or inflammatory conditions like perimyocarditis before proceeding with ablation to ensure the appropriateness and safety of the intervention.

The CHA_2_DS_2_-VA score is a reliable risk scale for prescribing oral anticoagulants (OAC) [88]. A CHA_2_DS_2_-VA score of 2 or more is recommended as an indicator of elevated thromboembolic risk, guiding decisions on initiating oral anticoagulation, while a CHA_2_DS_2_-VA score of 1 should also be taken into consideration for the initiation of oral anticoagulation, according to the most updated ESC guidelines in AF [88]. Patients receiving OAC should not participate in sports with direct bodily contact and are prone to trauma [89]. In cases of drug therapy failure, or an inability to receive medication, athletes would be recommended to procced with catheter ablation by pulmonary vein isolation (PVI) [90]. Patients should not participate in sports activities for at least 1 month after a successful ablation, resuming only if there are no recurrences of AF within this time period [11]. It is important to note that PVI ablation does not ensure the absence of non-pulmonary vein-dependent AF recurrences in the future. Specifically, PVI targets AF triggers originating from the pulmonary veins, but AF can recur due to triggers outside of these areas, including ectopic activity from the posterior left atrium, superior vena cava, or the coronary sinus, or from other mechanisms of the structural remodeling of the atria, unrelated to the pulmonary veins, such as fibrosis, inflammation, or electrical heterogeneity [91]. Given this complexity, recurrences may happen despite successful PVI, and it is often difficult to pinpoint specific triggers or predict their future behavior. In such cases, additional or alternative ablation strategies, such as targeting non-PV foci or substrate modification (e.g., posterior wall ablation or fractionated electrograms), may be considered, though there is no single definitive approach for these more complex cases. This uncertainty reflects the challenges in the long-term management of AF, as the mechanisms driving the arrhythmia are multifaceted and often not fully understood.

**Figure 3:**
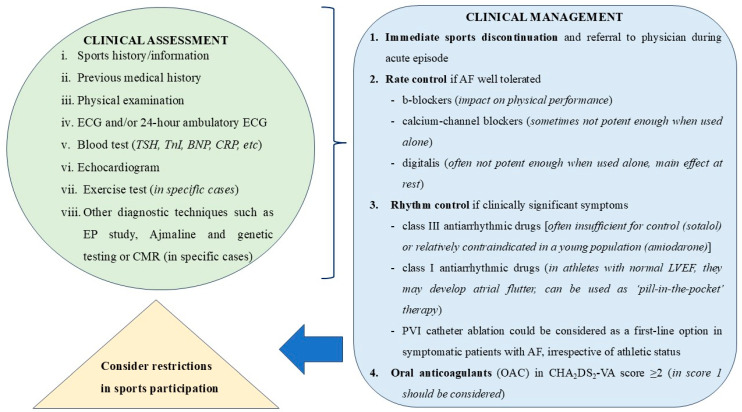
A proposed algorithm of clinical assessment and therapeutic strategy of an elite athlete with AF.

In conclusion, in the latest ESC and AHA guidelines in AF, physical activity and risk factor optimization are cornerstones for AF management before anticoagulation and rate or rhythm control. Still, there are no specific guidelines of exercise training for elite and non-elite athletes with AF, but only some consensus and recommendations. Below in Figure 3, we present a comprehensive evaluation of and management approach for an elite athlete with AF.

## 5. Conclusions

AF is a complex syndrome in elite athletes with multiple knowledge gaps, including pathophysiological mechanisms, such as alterations of autonomic system, systemic inflammation, and LA enlargement and fibrosis. Elite athletes with AF require a comprehensive clinical evaluation and risk factor optimization, similar to that for nonathletes. Although anticoagulation and the rate or rhythm control are cornerstones for AF management, there are still no specific guidelines for this subgroup. As a result, more studies are necessary for the further comprehension of the pathophysiological mechanisms and the establishment of individualized management algorithms.

## References

[1] Abed H.S., Wittert G.A., Leong D.P., Shirazi M.G., Bahrami B., Middeldorp M.E., Lorimer M.F., Lau D.H., Antic N.A., Brooks A.G. (2013). Effect of weight reduction and cardiometabolic risk factor management on symptom burden and severity in patients with atrial fibrillation: A randomized clinical trial. JAMA.

[2] Lee D.C., Sui X., Artero E.G., Lee I.M., Church T.S., McAuley P.A., Stanford F.C., Kohl H.W., Blair S.N. (2011). Long-term effects of changes in cardiorespiratory fitness and body mass index on all-cause and cardiovascular disease mortality in men: The Aerobics Center Longitudinal Study. Circulation.

[3] Lee D.C., Pate R.R., Lavie C.J., Sui X., Church T.S., Blair S.N. (2014). Leisure-time running reduces all-cause and cardiovascular mortality risk. J. Am. Coll. Cardiol..

[4] Turagam M.K., Velagapudi P., Kocheril A.G. (2012). Atrial fibrillation in athletes. Am. J. Cardiol..

[5] Abdulla J., Nielsen J.R. (2009). Is the risk of atrial fibrillation higher in athletes than in the general population? A systematic review and meta-analysis. Europace.

[6] Wilhelm M., Roten L., Tanner H., Wilhelm I., Schmid J.P., Saner H. (2011). Atrial remodeling, autonomic tone, and lifetime training hours in nonelite athletes. Am. J. Cardiol..

[7] Mont L., Tamborero D., Elosua R., Molina I., Coll-Vinent B., Sitges M., Vidal B., Scalise A., Tejeira A., Berruezo A. (2008). Physical activity, height, and left atrial size are independent risk factors for lone atrial fibrillation in middle-aged healthy individuals. Europace.

[8] Myrstad M., Nystad W., Graff-Iversen S., Thelle D.S., Stigum H., Aarønæs M., Ranhoff A.H. (2014). Effect of years of endurance exercise on risk of atrial fibrillation and atrial flutter. Am. J. Cardiol..

[9] Elliott A.D., Mahajan R., Pathak R.K., Lau D.H., Sanders P. (2016). Exercise Training and Atrial Fibrillation: Further Evidence for the Importance of Lifestyle Change. Circulation.

[10] Pelliccia A., Fagard R., Bjørnstad H.H., Anastassakis A., Arbustini E., Assanelli D., Biffi A., Borjesson M., Carrè F., Corrado D. (2005). Recommendations for competitive sports participation in athletes with cardiovascular disease: A consensus document from the Study Group of Sports Cardiology of the Working Group of Cardiac Rehabilitation and Exercise Physiology and the Working Group of Myocardial and Pericardial Diseases of the European Society of Cardiology. Eur. Heart J..

[11] Pelliccia A., Sharma S., Gati S., Bäck M., Börjesson M., Caselli S., Collet J.P., Corrado D., Drezner J.A., Halle M. (2021). 2020 ESC Guidelines on sports cardiology and exercise in patients with cardiovascular disease. Eur. Heart J..

[12] Estes N.A.M., Madias C. (2017). Atrial Fibrillation in Athletes: A Lesson in the Virtue of Moderation. JACC Clin. Electrophysiol..

[13] Sanchis-Gomar F., Perez-Quilis C., Lippi G., Cervellin G., Leischik R., Löllgen H., Serrano-Ostáriz E., Lucia A. (2017). Atrial fibrillation in highly trained endurance athletes—Description of a syndrome. Int. J. Cardiol..

[14] Andersen K., Farahmand B., Ahlbom A., Held C., Ljunghall S., Michaëlsson K., Sundström J. (2013). Risk of arrhythmias in 52 755 long-distance cross-country skiers: A cohort study. Eur. Heart J..

[15] Mozaffarian D., Furberg C.D., Psaty B.M., Siscovick D. (2008). Physical activity and incidence of atrial fibrillation in older adults: The cardiovascular health study. Circulation.

[16] Ofman P., Khawaja O., Rahilly-Tierney C.R., Peralta A., Hoffmeister P., Reynolds M.R., Gaziano J.M., Djousse L. (2013). Regular physical activity and risk of atrial fibrillation: A systematic review and meta-analysis. Circ. Arrhythm. Electrophysiol..

[17] Mohanty S., Mohanty P., Tamaki M., Natale V., Gianni C., Trivedi C., Gokoglan Y., DIBiase L., Natale A. (2016). Differential Association of Exercise Intensity with Risk of Atrial Fibrillation in Men and Women: Evidence from a Meta-Analysis. J. Cardiovasc. Electrophysiol..

[18] Petrungaro M., Fusco L., Cavarretta E., Scarà A., Borrelli A., Romano S., Petroni R., D’Ascenzi F., Sciarra L. (2023). Long-Term Sports Practice and Atrial Fibrillation: An Updated Review of a Complex Relationship. J. Cardiovasc. Dev. Dis..

[19] Morseth B., Graff-Iversen S., Jacobsen B.K., Jørgensen L., Nyrnes A., Thelle D.S., Vestergaard P., Løchen M.L. (2016). Physical activity, resting heart rate, and atrial fibrillation: The Tromsø Study. Eur. Heart J..

[20] Sciarra L., Cavarretta E., Siciliani S., Sette A., Scarà A., Grieco D., DERuvo E., Palamà Z., Nesti M., Romano S. (2022). Managing athletes with palpitations of unknown origin with an external loop recorder: A cohort study. J. Sports Med. Phys. Fitness.

[21] La Gerche A., Schmied C.M. (2013). Atrial fibrillation in athletes and the interplay between exercise and health. Eur. Heart J..

[22] Johansen K.R., Ranhoff A.H., Sørensen E., Nes B.M., Heitmann K.A., Apelland T., Bucher Sandbakk S., Wilsgaard T., Løchen M.L., Thelle D.S. (2022). Risk of atrial fibrillation and stroke among older men exposed to prolonged endurance sport practice: A 10-year follow-up. The Birkebeiner Ageing Study and the Tromsø Study. Open Heart.

[23] Svedberg N., Sundström J., James S., Hållmarker U., Hambraeus K., Andersen K. (2019). Long-Term Incidence of Atrial Fibrillation and Stroke Among Cross-Country Skiers. Circulation.

[24] Boraita A., Santos-Lozano A., Heras M.E., González-Amigo F., López-Ortiz S., Villacastín J.P., Lucia A. (2018). Incidence of Atrial Fibrillation in Elite Athletes. JAMA Cardiol..

[25] Newman W., Parry-Williams G., Wiles J., Edwards J., Hulbert S., Kipourou K., Papadakis M., Sharma R., O’Driscoll J. (2021). Risk of atrial fibrillation in athletes: A systematic review and meta-analysis. Br. J. Sports Med..

[26] Wernhart S., Halle M. (2015). Atrial fibrillation and long-term sports practice: Epidemiology and mechanisms. Clin. Res. Cardiol..

[27] Calvo N., Ramos P., Montserrat S., Guasch E., Coll-Vinent B., Domenech M., Bisbal F., Hevia S., Vidorreta S., Borras R. (2016). Emerging risk factors and the dose-response relationship between physical activity and lone atrial fibrillation: A prospective case-control study. Europace.

[28] Drca N., Larsson S.C., Grannas D., Jensen-Urstad M. (2023). Elite female endurance athletes are at increased risk of atrial fibrillation compared to the general population: A matched cohort study. Br. J. Sports Med..

[29] Myrstad M., Johansen K.R., Sørensen E., Løchen M.L., Ranhoff A.H., Morseth B. (2024). Atrial fibrillation in female endurance athletes. Eur. J. Prev. Cardiol..

[30] Wilhelm M., Roten L., Tanner H., Wilhelm I., Schmid J.P., Saner H. (2011). Gender differences of atrial and ventricular remodeling and autonomic tone in nonelite athletes. Am. J. Cardiol..

[31] Sanchis L., Sanz-de La Garza M., Bijnens B., Giraldeau G., Grazioli G., Marin J., Gabrielli L., Montserrat S., Sitges M. (2017). Gender influence on the adaptation of atrial performance to training. Eur. J. Sport Sci..

[32] Shoemaker M.B., Shah R.L., Roden D.M., Perez M.V. (2020). How Will Genetics Inform the Clinical Care of Atrial Fibrillation?. Circ. Res..

[33] Norby F.L., Benjamin E.J., Alonso A., Chugh S.S. (2021). Racial and Ethnic Considerations in Patients with Atrial Fibrillation: JACC Focus Seminar 5/9. J. Am. Coll. Cardiol..

[34] Gomez S.E., Fazal M., Nunes J.C., Shah S., Perino A.C., Narayan S.M., Tamirisa K.P., Han J.K., Rodriguez F., Baykaner T. (2023). Racial, ethnic, and sex disparities in atrial fibrillation management: Rate and rhythm control. J. Interv. Card. Electrophysiol..

[35] Chalazan B., Freeth E., Mohajeri A., Ramanathan K., Bennett M., Walia J., Halperin L., Roston T., Lazarte J., Hegele R.A. (2023). Genetic testing in monogenic early-onset atrial fibrillation. Eur. J. Hum. Genet..

[36] Yoneda Z.T., Anderson K.C., Quintana J.A., O’Neill M.J., Sims R.A., Glazer A.M., Shaffer C.M., Crawford D.M., Stricker T., Ye F. (2021). Early-Onset Atrial Fibrillation and the Prevalence of Rare Variants in Cardiomyopathy and Arrhythmia Genes. JAMA Cardiol..

[37] Hateley S., Lopez-Izquierdo A., Jou C.J., Cho S., Schraiber J.G., Song S., Maguire C.T., Torres N., Riedel M., Bowles N.E. (2021). The history and geographic distribution of a KCNQ1 atrial fibrillation risk allele. Nat. Commun..

[38] Lampert R., Chung E.H., Ackerman M.J., Arroyo A.R., Darden D., Deo R., Dolan J., Etheridge S.P., Gray B.R., Harmon K.G. (2024). 2024 HRS expert consensus statement on arrhythmias in the athlete: Evaluation, treatment, and return to play. Heart Rhythm..

[39] https://www.wada-ama.org/sites/default/files/2022-01/2022list_final_en_0.pdf.

[40] Lau D.H., Stiles M.K., John B., Young G.D., Sanders P. (2007). Atrial fibrillation and anabolic steroid abuse. Int. J. Cardiol..

[41] Seifert S.M., Schaechter J.L., Hershorin E.R., Lipshultz S.E. (2011). Health effects of energy drinks on children, adolescents, and young adults. Pediatrics.

[42] Di Rocco J.R., During A., Morelli P.J., Heyden M., Biancaniello T.A. (2011). Atrial fibrillation in healthy adolescents after highly caffeinated beverage consumption: Two case reports. J. Med. Case Rep..

[43] Steinke L., Lanfear D.E., Dhanapal V., Kalus J.S. (2009). Effect of “energy drink” consumption on hemodynamic and electrocardiographic parameters in healthy young adults. Ann. Pharmacother..

[44] Elliott A.D., Mahajan R., Lau D.H., Sanders P. (2016). Atrial Fibrillation in Endurance Athletes: From Mechanism to Management. Cardiol. Clin..

[45] Nattel S., Harada M. (2014). Atrial remodeling and atrial fibrillation: Recent advances and translational perspectives. J. Am. Coll. Cardiol..

[46] La Gerche A., Inder W.J., Roberts T.J., Brosnan M.J., Heidbuchel H., Prior D.L. (2015). Relationship between Inflammatory Cytokines and Indices of Cardiac Dysfunction following Intense Endurance Exercise. PLoS ONE.

[47] Sugama K., Suzuki K., Yoshitani K., Shiraishi K., Miura S., Yoshioka H., Mori Y., Kometani T. (2015). Changes of thioredoxin, oxidative stress markers, inflammation and muscle/renal damage following intensive endurance exercise. Exerc. Immunol. Rev..

[48] Aschar-Sobbi R., Izaddoustdar F., Korogyi A.S., Wang Q., Farman G.P., Yang F., Yang W., Dorian D., Simpson J.A., Tuomi J.M. (2015). Increased atrial arrhythmia susceptibility induced by intense endurance exercise in mice requires TNFα. Nat. Commun..

[49] Baggish A.L. (2015). Mechanisms underlying the cardiac benefits of exercise: Still running in the dark. Trends Cardiovasc. Med..

[50] Pelliccia A., Maron B.J., Di Paolo F.M., Biffi A., Quattrini F.M., Pisicchio C., Roselli A., Caselli S., Culasso F. (2005). Prevalence and clinical significance of left atrial remodeling in competitive athletes. J. Am. Coll. Cardiol..

[51] Turagam M.K., Flaker G.C., Velagapudi P., Vadali S., Alpert M.A. (2015). Atrial Fibrillation in Athletes: Pathophysiology, Clinical Presentation, Evaluation and Management. J. Atr. Fibrillation.

[52] Mont L., Elosua R., Brugada J. (2009). Endurance sport practice as a risk factor for atrial fibrillation and atrial flutter. Europace.

[53] Lindsay M.M., Dunn F.G. (2007). Biochemical evidence of myocardial fibrosis in veteran endurance athletes. Br. J. Sports Med..

[54] Wilson M., O’Hanlon R., Prasad S., Deighan A., Macmillan P., Oxborough D., Godfrey R., Smith G., Maceira A., Sharma S. (2011). Diverse patterns of myocardial fibrosis in lifelong, veteran endurance athletes. J. Appl. Physiol..

[55] Swanson D.R. (2006). Atrial fibrillation in athletes: Implicit literature-based connections suggest that overtraining and subsequent inflammation may be a contributory mechanism. Med. Hypotheses.

[56] Bettoni M., Zimmermann M. (2002). Autonomic tone variations before the onset of paroxysmal atrial fibrillation. Circulation.

[57] Grimsmo J., Grundvold I., Maehlum S., Arnesen H. (2010). High prevalence of atrial fibrillation in long-term endurance cross-country skiers: Echocardiographic findings and possible predictors—A 28–30 years follow-up study. Eur. J. Cardiovasc. Prev. Rehabil..

[58] Carpenter A., Frontera A., Bond R., Duncan E., Thomas G. (2015). Vagal atrial fibrillation: What is it and should we treat it?. Int. J. Cardiol..

[59] Grundvold I., Skretteberg P.T., Liestøl K., Erikssen G., Engeseth K., Gjesdal K., Kjeldsen S.E., Arnesen H., Erikssen J., Bodegard J. (2013). Low heart rates predict incident atrial fibrillation in healthy middle-aged men. Circ. Arrhythm. Electrophysiol..

[60] Swanson D.R. (2008). Running, esophageal acid reflux, and atrial fibrillation: A chain of events linked by evidence from separate medical literatures. Med. Hypotheses.

[61] Collings K.L., Pierce Pratt F., Rodriguez-Stanley S., Bemben M., Miner P.B. (2003). Esophageal reflux in conditioned runners, cyclists, and weightlifters. Med. Sci. Sports Exerc..

[62] Soffer E.E., Merchant R.K., Duethman G., Launspach J., Gisolfi C., Adrian T.E. (1993). Effect of graded exercise on esophageal motility and gastroesophageal reflux in trained athletes. Dig. Dis. Sci..

[63] Choi S.C., Choi S.J., Kim J.A., Kim T.H., Nah Y.H., Yazaki E., Evans D.F. (2001). The role of gastrointestinal endoscopy in long-distance runners with gastrointestinal symptoms. Eur. J. Gastroenterol. Hepatol..

[64] Kunz J.S., Hemann B., Edwin Atwood J., Jackson J., Wu T., Hamm C. (2009). Is there a link between gastroesophageal reflux disease and atrial fibrillation?. Clin. Cardiol..

[65] Proietti M., Boriani G., Laroche C., Diemberger I., Popescu M.I., Rasmussen L.H., Sinagra G., Dan G.A., Maggioni A.P., Tavazzi L. (2017). Self-reported physical activity and major adverse events in patients with atrial fibrillation: A report from the EURObservational Research Programme Pilot Survey on Atrial Fibrillation (EORP-AF) General Registry. Europace.

[66] Nortamo S., Ukkola O., Lepojärvi S., Kenttä T., Kiviniemi A., Junttila J., Huikuri H., Perkiömäki J. (2017). Association of sST2 and hs-CRP levels with new-onset atrial fibrillation in coronary artery disease. Int. J. Cardiol..

[67] Fashanu O.E., Norby F.L., Aguilar D., Ballantyne C.M., Hoogeveen R.C., Chen L.Y., Soliman E.Z., Alonso A., Folsom A.R. (2017). Galectin-3 and incidence of atrial fibrillation: The Atherosclerosis Risk in Communities (ARIC) study. Am. Heart J..

[68] McManus D.D., Tanriverdi K., Lin H., Esa N., Kinno M., Mandapati D., Tam S., Okike O.N., Ellinor P.T., Keaney J.F. (2015). Plasma microRNAs are associated with atrial fibrillation and change after catheter ablation (the miRhythm study). Heart Rhythm..

[69] Hättasch R., Spethmann S., de Boer R.A., Ruifrok W.P., Schattke S., Wagner M., Schroeckh S., Durmus T., Schimke I., Sanad W. (2014). Galectin-3 increase in endurance athletes. Eur. J. Prev. Cardiol..

[70] Roca E., Nescolarde L., Lupón J., Barallat J., Januzzi J.L., Liu P., Cruz Pastor M., Bayes-Genis A. (2017). The Dynamics of Cardiovascular Biomarkers in non-Elite Marathon Runners. J. Cardiovasc. Transl. Res..

[71] Baggish A.L., Hale A., Weiner R.B., Lewis G.D., Systrom D., Wang F., Wang T.J., Chan S.Y. (2011). Dynamic regulation of circulating microRNA during acute exhaustive exercise and sustained aerobic exercise training. J. Physiol..

[72] Luo N., Merrill P., Parikh K.S., Whellan D.J., Piña I.L., Fiuzat M., Kraus W.E., Kitzman D.W., Keteyian S.J., O’Connor C.M. (2017). Exercise Training in Patients with Chronic Heart Failure and Atrial Fibrillation. J. Am. Coll. Cardiol..

[73] Rienstra M., Hobbelt A.H., Alings M., Tijssen J.G.P., Smit M.D., Brügemann J., Geelhoed B., Tieleman R.G., Hillege H.L., Tukkie R. (2018). Targeted therapy of underlying conditions improves sinus rhythm maintenance in patients with persistent atrial fibrillation: Results of the RACE 3 trial. Eur. Heart J..

[74] Malmo V., Nes B.M., Amundsen B.H., Tjonna A.E., Stoylen A., Rossvoll O., Wisloff U., Loennechen J.P. (2016). Aerobic Interval Training Reduces the Burden of Atrial Fibrillation in the Short Term: A Randomized Trial. Circulation.

[75] Kemi O.J., Wisløff U. (2010). Mechanisms of exercise-induced improvements in the contractile apparatus of the mammalian myocardium. Acta Physiol..

[76] Pathak R.K., Middeldorp M.E., Lau D.H., Mehta A.B., Mahajan R., Twomey D., Alasady M., Hanley L., Antic N.A., McEvoy R.D. (2014). Aggressive risk factor reduction study for atrial fibrillation and implications for the outcome of ablation: The ARREST-AF cohort study. J. Am. Coll. Cardiol..

[77] Elliott A.D., Verdicchio C.V., Mahajan R., Middeldorp M.E., Gallagher C., Mishima R.S., Hendriks J.M.L., Pathak R.K., Thomas G., Lau D.H. (2023). An Exercise and Physical Activity Program in Patients with Atrial Fibrillation: The ACTIVE-AF Randomized Controlled Trial. JACC Clin. Electrophysiol..

[78] AbuElkhair A., Boidin M., Buckley B.J.R., Lane D.A., Williams N.H., Thijssen D., Lip G.Y.H., Barraclough D.L. (2023). Effects of different exercise types on quality of life for patients with atrial fibrillation: A systematic review and meta-analysis. J. Cardiovasc. Med..

[79] Moorman A.J., Dean L.S., Yang E., Drezner J.A. (2021). Cardiovascular Risk Assessment in the Older Athlete. Sports Health..

[80] Harmon K.G., Zigman M., Drezner J.A. (2015). The effectiveness of screening history, physical exam, and ECG to detect potentially lethal cardiac disorders in athletes: A systematic review/meta-analysis. J. Electrocardiol..

[81] Sharma S., Drezner J.A., Baggish A., Papadakis M., Wilson M.G., Prutkin J.M., La Gerche A., Ackerman M.J., Borjesson M., Salerno J.C. (2018). International recommendations for electrocardiographic interpretation in athletes. Eur. Heart J..

[82] Niederseer D., Rossi V.A., Kissel C., Scherr J., Caselli S., Tanner F.C., Bohm P., Schmied C. (2020). Role of echocardiography in screening and evaluation of athletes. Heart.

[83] Jewson J.L., Orchard J.W., Semsarian C., Fitzpatrick J., La Gerche A., Orchard J.J. (2022). Use of a smartphone electrocardiogram to di-agnose arrhythmias during exercise in athletes: A case series. Eur. Heart J. Case Rep..

[84] Steinberg J.S., Varma N., Cygankiewicz I., Aziz P., Balsam P., Baranchuk A., Cantillon D.J., Dilaveris P., Dubner S.J., El-Sherif N. (2017). ISHNE-HRS expert consensus statement on ambulatory ECG and external cardiac monitoring/telemetry. Heart Rhythm..

[85] Zipes D.P., Link M.S., Ackerman M.J., Kovacs R.J., Myerburg R.J., Estes N.A.M. (2015). Eligibility and Disqualification Recommendations for Competitive Athletes with Cardiovascular Abnormalities: Task Force 9: Arrhythmias and Conduction Defects: A Scientific Statement from the American Heart Association and American College of Cardiology. J. Am. Coll. Cardiol..

[86] Koldenhof T., Wijtvliet P.E.P.J., Pluymaekers N.A.H.A., Rienstra M., Folkeringa R.J., Bronzwaer P., Elvan A., Elders J., Tukkie R., Luermans J.G.L.M. (2022). Rate control drugs differ in the prevention of progression of atrial fibrillation. Europace.

[87] Alboni P., Botto G.L., Baldi N., Luzi M., Russo V., Gianfranchi L., Marchi P., Calzolari M., Solano A., Baroffio R. (2004). Outpatient treatment of recent-onset atrial fibrillation with the “pill-in-the-pocket” approach. N. Engl. J. Med..

[88] Van Gelder I.C., Rienstra M., Bunting K.V., Casado-Arroyo R., Caso V., Crijns H.J.G.M., De Potter T.J.R., Dwight J., Guasti L., Hanke T. (2024). ESC Guidelines for the management of atrial fibrillation developed in collaboration with the European Association for Cardio-Thoracic Surgery (EACTS). Eur. Heart J..

[89] Steffel J., Verhamme P., Potpara T.S., Albaladejo P., Antz M., Desteghe L., Haeusler K.G., Oldgren J., Reinecke H., Roldan-Schilling V. (2018). The 2018 European Heart Rhythm Association Practical Guide on the use of non-vitamin K antagonist oral anticoagulants in patients with atrial fibrillation. Eur. Heart J..

[90] Calkins H., Hindricks G., Cappato R., Kim Y.H., Saad E.B., Aguinaga L., Akar J.G., Badhwar V., Brugada J., Camm J. (2017). 2017 HRS/EHRA/ECAS/APHRS/SOLAECE expert consensus statement on catheter and surgical ablation of atrial fibrillation. Heart Rhythm..

[91] Kawai S., Mukai Y., Inoue S., Yakabe D., Nagaoka K., Sakamoto K., Takase S., Chishaki A., Tsutsui H. (2019). Non-Pulmonary Vein Triggers of Atrial Fibrillation Are Likely to Arise from Low-Voltage Areas in the Left Atrium. Sci. Rep..

